# Comprehensive Luciferase-Based Reporter Gene Assay Reveals Previously Masked Up-Regulatory Effects of miRNAs

**DOI:** 10.3390/ijms150915592

**Published:** 2014-09-03

**Authors:** Danae Campos-Melo, Cristian A. Droppelmann, Kathryn Volkening, Michael J. Strong

**Affiliations:** 1 Molecular Medicine Group, Robarts Research Institute, Western University, London, ON N6A 5B7, Canada; E-Mails: dmaribel@uwo.ca (D.C.-M.); cdroppel@uwo.ca (C.A.D.); kvolkening@robarts.ca (K.V.); 2 Department of Clinical Neurological Sciences, Schulich School of Medicine and Dentistry, Western University, London, ON N6A 3K7, Canada; 3 Department of Pathology, Western University, London, ON N6A 3K7, Canada

**Keywords:** reporter gene assay, miRNA, normalization, NEFL (neurofilament)

## Abstract

MicroRNAs (miRNAs) are small non-coding RNAs that regulate the majority of the transcriptome at a post-transcriptional level. Because of this critical role, it is important to ensure that the assays used to determine their functionality are robust and reproducible. Typically, the reporter gene assay in cell-based systems has been the first-line method to study miRNA functionality. In order to overcome some of the potential errors in interpretation that can be associated with this assay, we have developed a detailed protocol for the luciferase reporter gene assay that has been modified for miRNAs. We demonstrate that normalization against the effect of the miRNA and cellular factors on the luciferase coding sequence is essential to obtain the specific impact of the miRNA on the 3'UTR (untranslated region) target. Our findings suggest that there is a real possibility that the roles for miRNA in transcriptome regulation may be misreported due to inaccurate normalization of experimental data and also that up-regulatory effects of miRNAs are not uncommon in cells. We propose to establish this comprehensive method as standard for miRNA luciferase reporter assays to avoid errors and misinterpretations in the functionality of miRNAs.

## 1. Introduction

MicroRNAs (miRNAs) are small non-coding RNAs predicted to post-transcriptionally regulate the majority of the transcriptome [1]. These 20–24 nucleotide-long RNAs have been involved in numerous physiological processes such as development and differentiation [2], and have been linked to many human disorders including cancer [3] and neurodegenerative disease [4].

MiRNAs are recognized mainly as negative post-transcriptional regulators of gene expression. They exert their function by association to miRNA recognition elements (MREs), predominantly within the 3' untranslated region (UTR) of mRNAs. In most cases, miRNA binding to mRNA results in the repression of the transcript by one of two mechanisms: mRNA cleavage or translational repression [5].

To understand the role of miRNAs in different physiological processes, it is important to utilize experimental approaches that allow for the detection of fine miRNA-induced alterations in mRNA expression. Currently, the functional relevance of MREs is extensively assessed using reporter gene assays in cell-based systems. In the miRNA-adapted version of this assay, reporter activity is an indicator of miRNA capacity in regulating the reporter expression *in vitro*. Cells are co-transfected with the miRNA and a plasmid containing a *Firefly* or *Renilla* luciferase coding sequence upstream of an mRNA 3'UTR from the gene of interest. If the mRNA 3'UTR is a target of the miRNA, the luminescence variation will be altered, a reflection of the changes in the transcript’s stability and/or translation efficiency.

Recently, we profiled miRNAs from amyotrophic lateral sclerosis (ALS) spinal cord tissues and used miRNA reporter gene assays to test the functionality of dysregulated miRNAs [6]. We observed that the controls and the normalization methods most commonly reported in these assays do not take into consideration the possibility of direct and/or indirect effects of the miRNAs on the luciferase mRNA.

Here, we present a comprehensive miRNA reporter gene assay protocol that includes a rigorous normalization of the data. We show that incomplete normalization can lead to erroneous conclusions, not only to the magnitude of the miRNA expression regulation, but more importantly to the type of miRNA regulation (up- or down-regulation). Interestingly, these results suggest that miRNA up-regulatory effects are not uncommon and could reveal previously undetected roles for miRNAs in regulating the transcriptome.

## 2. Results

Currently, the luciferase reporter gene assay is extensively used to evaluate the functional relevance of miRNAs. We have observed that studies in the miRNA field lack rigorous normalization of the reporter activity data, generally only showing a transfection control (such as *Renilla* luciferase) and a control without miRNA or a non- targeting miRNA negative control. These types of analyses do not consider the possibility of indirect effects of miRNAs on the luciferase transcript and/or the existence of functional MREs in the luciferase protein coding region [7,8,9]. Therefore, it is important to consider that, in addition to their putative effects on their mRNAs targets, miRNAs and other cellular factors could potentially regulate luciferase mRNA, critically affecting its activity. It is important to note that, while miRNAs and other factors could affect both *Firefly* and *Renilla* luciferase mRNA levels, it is the regulation of the luciferase linked to the mRNA 3'UTR (*Firefly* in our study) that is relevant for the normalization of the reporter gene assay data. Any miRNA or cellular effect over the control luciferase (*Renilla* in our study) will be cancelled due to the normalization.

**Figure 1:**
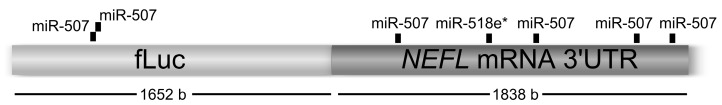
miRNA recognition elements (MREs) for miR-507 and miR-518e* within *Firefly* luciferase linked to the neurofilament (*NEFL*) mRNA 3'UTR.

We performed reporter assays by transfecting HEK293T cells with a plasmid containing *Firefly* and *Renilla* luciferase coding sequences (pmirGLO) or the same vector harboring the low molecular weight neurofilament (*NEFL*) mRNA 3'UTR linked to the 3'-end of the *Firefly* luciferase (pmirGLO-*NEFL* 3'UTR). *Renilla* luciferase was used as transfection control. In these experiments, the independent effect of three miRNAs, miR-507, miR-518e* and miR-let-7a (negative control) were studied. Analyses using miRanda algorithm (see experimental section) showed that miR-507 has four MREs and miR-518e* has one MRE within the *NEFL* mRNA 3'UTR. Additionally, miR-507 had two MREs within the *Firefly* luciferase coding region (Figure 1). Six wells of each condition were transfected to obtain the mean of luciferase activity of each experiment (for details, see Experimental Section).

After normalizing each sample’s *Firefly* luciferase activity value with its respective *Renilla* luciferase activity value (data not shown), we calculated the mean of the luciferase activity (Mean Luc) for each experiment (*n* = 3; Table 1). In this step of the analysis, we noticed that both miR-507 and miR-518e* down-regulated the luciferase activity when pmirGLO-*NEFL* 3'UTR was transfected. Interestingly, the negative control miR-let-7a also appeared to down-regulate the luciferase activity (Table 1, Mean Luc columns, rows b compared to rows a). We then analyzed the effect of each miRNA on the luciferase mRNA without the 3'UTR (pmirGLO transfection), and again found that miR-507, miR-518e* and miR-let-7a down-regulated the luciferase activity (Table 1, Mean Luc columns, rows d compared to rows c).

**Table 1:** Luciferase reporter assay data for miR-507, miR-518e* and miR-let-7a effects on *NEFL* 3'UTR.

miR-507 Transfection	Mean Luc			pmirGLO-*NEFL* 3'UTR + miR-507 pmirGLO-*NEFL* 3'UTR	pmirGLO+miR-507 pmirGLO	Luc Relative Variation
	Exp 1	Exp 2	Exp 3	A	B	A/B
(a) pmirGLO-*NEFL* 3'UTR + miR-507	2.20	2.21	2.23	0.40 ± 0.10 10^−2^	-	0.82 ± 0.02
(b) pmirGLO-*NEFL* 3'UTR	5.52	5.53	5.47			
(c) pmirGLO + miR-507	8.42	7.09	7.46	-	0.49 ± 0.12 10^−1^	
(d) pmirGLO	15.71	15.71	15.58			
miR-518e* Transfection	**Mean Luc**			pmirGLO-*NEFL* 3'UTR + miR-518* **pmirGLO-*NEFL* 3'UTR**	pmirGLO + miR-518e* **pmirGLO**	**Luc Relative Variation**
	**Exp 1**	**Exp 2**	**Exp 3**	**A**	**B**	**A/B**
(a) pmirGLO-*NEFL* 3'UTR + miR-518e*	2.82	2.74	2.82	0.83 ± 0.82 10^−2^	-	1.66 ± 0.02
(b) pmirGLO-*NEFL* 3'UTR	3.30	3.34	3.51			
(c) pmirGLO + miR-518e*	5.21	5.16	5.12	-	0.50 ± 0.19 10^−2^	
(d) pmirGLO	10.52	10.30	10.40			
**miR-let-7a Transfection**	**Mean Luc**			**pmirGLO-*NEFL* 3'UTR + miR-let-7a** **pmirGLO-*NEFL* 3'UTR**	**pmirGLO + miR-let-7a** **pmirGLO**	**Luc Relative Variation**
	**Exp 1**	**Exp 2**	**Exp 3**	**A**	**B**	**A/B**
(a) pmirGLO-*NEFL* 3'UTR + miR-let-7a	4.42	3.98	4.48	0.74 ± 0.14 10^−1^	-	1.05 ± 0.03
(b) pmirGLO-*NEFL* 3'UTR	5.80	5.70	5.97			
(c) pmirGLO + miR-let7a	9.89	9.96	9.07	-	0.70 ± 0.10 10^−1^	
(d) pmirGLO	13.90	13.85	13.52			

Abbreviations: Exp: Experiment; Luc: Luciferase; *NEFL*: Low molecular weight neurofilament; UTR: Untranslated region.

**Figure 2:**
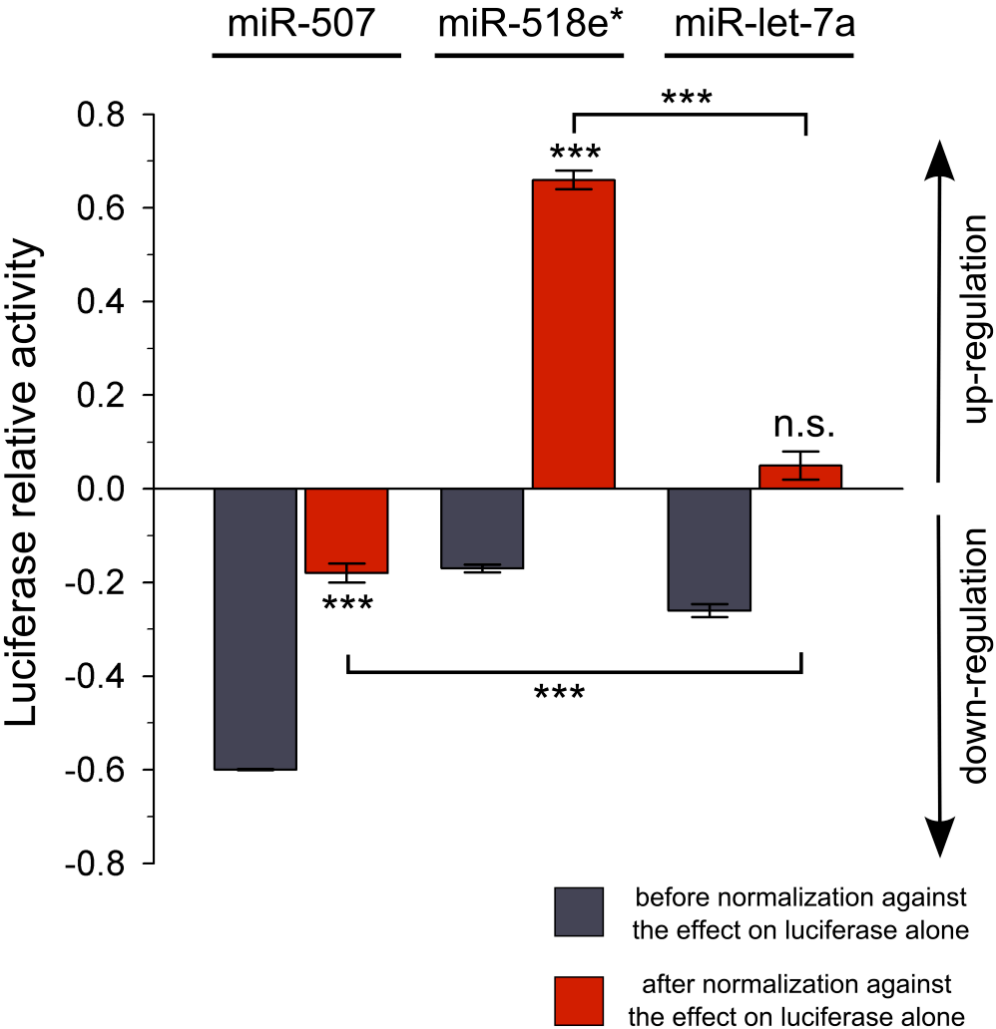
Luciferase reporter assay using miR-507, miR-518e* or miR-let-7a and the *NEFL* 3'UTR. Data from reporter assays before (gray) and after the normalization (red) against the effect of the miRNAs on the *Firefly* luciferase reporter alone are shown. Data from Table 1 are expressed as positive values for up-regulation and negative values for down-regulation (X-1). Experiments were performed in triplicate. Results are shown as mean ± SEM. *t*-test was performed to compare the effect of each miRNA on the *NEFL* 3'UTR with the effect on the *Firefly* luciferase (*** = *p* < 0.001; n.s. = not significant). Comparison between miR-507 or miR-518e* with miR-let-7a negative control was performed using ANOVA, Newman-Keuls test (*** = *p* < 0.001).

In a second step of normalization, we calculated the effect of miR-507, miR-518e* and miR-let-7a on the *Firefly* luciferase with or without the *NEFL* mRNA 3'UTR (Table 1, columns A and B; Figure 2). This normalization is critical to obtain the specific effect of the miRNA over the *NEFL* mRNA 3'UTR.

**Figure 3:**
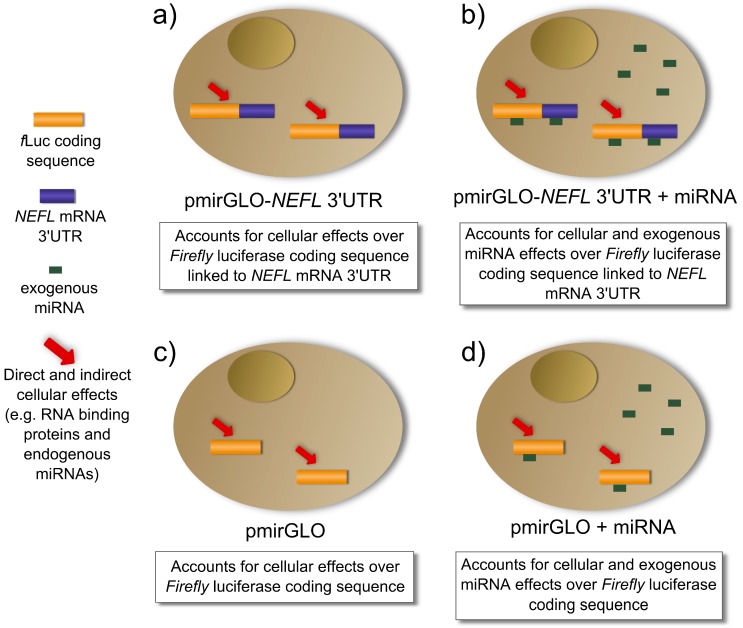
Cellular and exogenous miRNA effects regulating the luciferase activity in the reporter gene assay. (**a**,**b**) Cells transfected with luciferase with the *NEFL* mRNA 3'UTR; (**c**,**d**) Cells transfected with luciferase without 3'UTR.

As is described in Figure 3a, the transfection of pmirGLO-*NEFL* 3'UTR accounts for cellular effects (e.g., RNA binding proteins, endogenous miRNAs, *etc.*) on the expression of luciferase linked to *NEFL* mRNA 3'UTR. The co-transfection of the pmirGLO-*NEFL* 3'UTR and the exogenous miRNA account for cellular and exogenous miRNA effects on the expression of luciferase linked to *NEFL* mRNA 3'UTR (Figure 3b). When the normalization of column A is performed (Table 1), cancellation of the cellular effects occurs, leaving only the exogenous miRNA effects on the expression of luciferase linked to *NEFL* mRNA 3'UTR

Figure 3c,d describe the controls, pmirGLO and pmirGLO plus the exogenous miRNA respectively, which account for the effects on luciferase expression without 3'UTR. When the normalization of column B is performed (Table 1), we obtain the effect of exogenous miRNA on luciferase expression without 3'UTR.

Thus, to obtain the specific effect of the exogenous miRNA on only *NEFL* mRNA 3'UTR, the effect of this miRNA over the luciferase coding sequence must be eliminated. This is achieved by dividing column A by B (Table 1, Luc relative variation column; Figure 2). Calculation of the relative variation in luciferase activity showed that only miR-507 down-regulated the activity of luciferase (0.82 ± 0.02, *p* < 0.001). Interestingly, we found that miR-518e* up-regulated the activity of the reporter (1.66 ± 0.02; *p* < 0.001). No relative changes in the activity of the reporter linked to the *NEFL* mRNA 3'UTR was found in cells transfected with miR-let-7a control miRNA (1.05 ± 0.03, *p* = 0.059).

Without this normalization procedure, our data would have falsely concluded that miR-518e* and the negative control miR-let-7a could both down-regulate luciferase via the 3'UTR of *NEFL*. Our results clearly demonstrate that performing incomplete controls or normalization on the data of the miRNA reporter gene assay without considering the impact of cellular factors and exogenous miRNAs on luciferase without 3'UTR could generate miscalculation of the relative variation in luciferase activity, leading to erroneous conclusions about miRNA functionality.

## 3. Discussion

The most extensive experimental approaches to determine functional relevance of predicted MREs are the electrophoretic mobility shift assay (EMSA) to detect interaction between miRNAs and their targets, and the reporter gene assay to study miRNA regulatory effects. Considering that the reporter gene assay is a unique functional tool to study miRNA-based regulation on mRNA expression, it is extremely important to perform appropriate data analysis to avoid inaccurate results and conclusions. Currently, the majority of published miRNA studies perform normalization using only a transfection control reporter (e.g., *Renilla* luciferase) and controls lacking exogenous miRNA or transfecting a miRNA negative control with luciferase linked to the 3'UTR as target. However, the effect of the exogenous miRNA and cellular factors on the luciferase coding sequence alone has not been included as a control, and as described here, must be considered. We demonstrate that normalization against the effect of the miRNA and cellular factors on the luciferase coding sequence alone is essential to obtaining the specific impact of the miRNA on the 3'UTR target. We propose this normalization method as standard for miRNA luciferase reporter assays. Therefore, errors and misinterpretations in miRNA functionality commonly observed using previous protocols can be avoided.

MiRNAs predominantly, but not exclusively, exert their functions through interaction with mRNA 3'UTRs [10]. There is evidence that miRNAs have functional MREs in 5'UTRs [11,12] and protein coding regions of mammalian transcripts [13,14]. In fact, Argonaute HITS-CLIP and PAR-CLIP studies have shown that almost half of all Argonaute binding sites are located in human protein coding regions [15,16]. In addition, it is well known that reporter gene assays reveal specific regulation of miRNAs on their targets that could be both directly mediated through interaction, and indirect for instance through the regulation of other factors involved in mRNA stability. For example, miR-375 down-regulates the expression of HuD, an RNA-binding protein that participates in mRNA stability and other regulatory processes, and thus governs the fate of many neuronal mRNAs [17] through an independent mechanism.

Prediction analysis showed MREs for both miR-507 and miR-518e* within the *NEFL* mRNA 3'UTR. The results of the reporter gene assay support the idea that both miRNAs regulate the reporter linked to the *NEFL* mRNA 3'UTR. After normalizing against both the transfection control (*Renilla* luciferase) and the control without the miRNA, miR-507 shows a down-regulatory effect on the luciferase linked to the *NEFL* mRNA 3'UTR that once normalized for effects on luciferase, is only half as strong as what it was originally detected to be before normalization. In fact, a manual search revealed two MREs for miR-507 within the *Firefly* coding sequence, and the reporter gene assay showed that miR-507 regulates *Firefly* luciferase. Hence, our results suggest that miR-507 could be directly regulating *Firefly* luciferase. However, it is important to consider that site accessibility and translation efficiency around MREs are two factors that might affect miRNA binding when MREs are located in coding regions [18]. Unlike miR-507, the regulation by miR-518e* changes from negative to positive after the normalization against the effect of miR-518e* over *Firefly* luciferase. Considering that miR-518e* does not have binding sites within the *Firefly* luciferase coding sequence, our reporter assay results suggest an indirect down-regulatory effect of miR-518e*. All of this evidence supports the idea that miRNAs might regulate *Firefly* luciferase in miRNA reporter gene assays, and as a consequence the effect of each miRNA on the luciferase mRNA itself must be included in the normalization of the data. It is important to note that while the reporter gene assay is a highly sensitive and reliable technique, using confirmatory and complementary methods to study the regulatory effects of miRNAs on their targets is greatly recommended. Studies of miRNA effects on mRNA and protein levels and the usage of MRE mutants to determine the specificity of these effects are all valuable approaches.

Although the actions of miRNAs on mRNA cleavage or translational repression are their more well-known roles, there is increasing evidence of miRNAs being involved in up-regulation of the mRNA expression [19,20,21]. For instance, in mammalian cells the translation of ribosomal proteins and other proteins involved in protein synthesis is regulated via the 5'TOP motif, which renders transcripts sensitive to stress signals. MiR-10a can interact with the 5'UTR, immediately downstream of the regulatory 5'TOP motif, and enhances the translation of ribosomal proteins [21]. Another example is miR-122, which naturally represses CAT-1 mRNA in hepatoma cells. However, in the presence of HuR protein, the repression of CAT-1 is removed due to the interaction of HuR with the CAT-1 mRNA 3'UTR. Thus, HuR acts as a modifier altering the ability of miRNAs to repress gene expression [22]. Recently, we reported a group of dysregulated miRNAs in ALS that regulate the expression of a reporter linked to the *NEFL* mRNA 3'UTR. We found that a significant group of miRNAs up-regulate the expression of luciferase linked to the *NEFL* mRNA 3'UTR [6]. The novel results shown in this paper and the data we published previously suggest that up-regulatory roles for miRNAs are not an uncommon phenomenon.

## 4. Experimental Section

### 4.1. Plasmid Construction, Cell Culture and Transfection

The reporter plasmid was constructed by inserting human low molecular weight neurofilament (*NEFL*) mRNA 3'UTR (1–1838; GenBank NM_006158) between NheI and SalI sites downstream of the *Firefly* luciferase gene in the pmirGLO vector (Promega, Madison, WI, USA).

HEK293T cells were maintained in Dulbecco’s modified Eagle’s medium (DMEM) containing 10% fetal bovine serum (FBS) and plated 24 h before transfection in 96-well plates at 9 × 10^3^ cells/well. Cells were transfected with 3.47 fmol of pmirGLO-*NEFL* 3'UTR or pmirGLO and 100 nM of pre-miRNAs (Life Technologies Inc., Burlington, ON, Canada) per well using Lipofectamine 2000 reagent (Life Technologies Inc.) according to the manufacturer’s instructions. The total amount of transfected nucleic acid was maintained constant at 350 ng per well by adding an empty vector without eukaryotic elements. Six wells of each specific condition were transfected to obtain the mean of luciferase activity of each experiment. All the experiments were performed in triplicate.

### 4.2. Luciferase Reporter Assay

Luciferase activity was measured 24 h after transfection using the Dual-Glo Luciferase Assay System (Promega; Madison, WI, USA) in a Luminometer (Turner Biosystems Luminometer, Promega) according to the manufacturer’s instructions. Normalization of the data included two sequential steps. (1) *Firefly* luciferase activity was normalized to *Renilla* luciferase activity to account for variations in the transfection efficiency among experiments. Normalized values out of the mean range of each experiment were eliminated, always maintaining at least four values from the six transfected wells; (2) The luciferase activity from each miRNA over the 3'UTR was normalized against the effects of the miRNA on luciferase mRNA without the 3'UTR to finally obtain the specific effect of the miRNA on the *NEFL* mRNA 3'UTR (for more detailed discussion see the results section). Values of relative variation in luciferase activity over 1 were considered up-regulation and values under 1 were considered down-regulation. Quantitative data of the reporter gene assay are presented as mean ± SEM. Student’s *t-*test was used to determine significant differences between the effect of each miRNA on the *NEFL* 3'UTR or the *Firefly* luciferase alone. Comparison between miR-507 or miR-518e* and miR-let-7a negative control was performed using ANOVA, Newman-Keuls test.

### 4.3. MiRNA Target Prediction

To determine miRNA recognition elements (MREs) within the *NEFL* mRNA 3'UTR, we used the miRanda algorithm [23]. *Firefly* and *Renilla* coding regions were examined manually in the prediction of MREs considering the criteria of the seed sequence [24].

## 5. Conclusions

Our results using the miRNA reporter gene assay protocol presented in this paper suggest that: (1) roles of miRNAs could be masked by miRNA effects on the reporter being revealed only through proper normalization of the data; (2) up-regulatory roles of miRNAs could be a common regulatory mechanism in cells; and (3) there could be unrevealed roles for miRNAs as regulatory elements of the transcriptome.
